# Case report: Genomic screening for inherited cardiac conditions in Ecuadorian mestizo relatives: Improving familial diagnose

**DOI:** 10.3389/fcvm.2022.1037370

**Published:** 2022-11-08

**Authors:** Santiago Cadena-Ullauri, Patricia Guevara-Ramirez, Viviana Ruiz-Pozo, Rafael Tamayo-Trujillo, Elius Paz-Cruz, Tatiana Sánchez Insuasty, Nieves Doménech, Adriana Alexandra Ibarra-Rodríguez, Ana Karina Zambrano

**Affiliations:** ^1^Centro de Investigación Genética y Genómica, Facultad de Ciencias de la Salud Eugenio Espejo, Universidad UTE, Quito, Ecuador; ^2^Cardióloga ecocardiografísta, Centros Médicos Especializados Cruz Roja Ecuatoriana, Quito, Ecuador; ^3^Instituto de Investigación Biomédica de A Coruña (INIBIC)-CIBERCV, Complexo Hospitalario Universitario A Coruña (CHUAC), Sergas, Universidad da Coruña (UDC), La Coruña, Spain; ^4^Grupo de Investigación Identificación Genética-IdentiGEN, FCEN, Universidad de Antioquia, Medellín, Colombia

**Keywords:** genomic, cardiovascular disease, case report, genetics, ancestral

## Abstract

**Introduction:**

Genomic screening is an informative and helpful tool for the clinical management of inherited conditions such as cardiac diseases. Cardiac-inherited diseases are a group of disorders affecting the heart, its system, function, and vasculature. Among the cardiac inherited abnormalities, one of the most common is Wolff-Parkinson-White syndrome. Similarly, hypertrophic cardiomyopathy is another common autosomal dominant inherited cardiac disease. Hypertrophic cardiomyopathy is associated with an increased incidence of Wolff-Parkinson-White syndrome; reports have suggested that it could be caused by a mutation in the protein-coding gene PRKAG2, which encodes a subunit of the AMP-activated protein kinase.

**Case presentation:**

A 37-year-old Ecuadorian male (Subject A) with familiar history of bradycardia, cardiac pacemaker implantation, and undiagnosed cardiac conditions began with episodes of tachycardia, dizziness, shortness of breath, and a feeling of fainting. He was diagnosed with hypertrophic myocardiopathy and Wolff Parkinson White preexcitation syndrome. Furthermore, his cousin's son, an 18-year-old Ecuadorian male (Subject B), started suffering from migraine and tachycardia at any time of the day. He was diagnosed with hypertrophic myocardiopathy; his electrocardiogram showed a systolic overload. Next-generation sequencing and ancestry analyses were performed. A c.905G>A p.(Arg302Gln) mutation in the gene PRKAG2 and a mainly European composition were identified in both subjects.

**Conclusion:**

Genetic testing is a valuable tool as it can provide important information regarding a disease, including its cause and consequences, not only for single individuals but to identify at-risk relatives. Furthermore, NGS results could guide the physician into targeted therapy. In the present case report, a missense pathogenic Arg302Gln mutation in the PRKAG2 gene has been identified in two related Ecuadorian Subjects diagnosed with hypertrophic myocardiopathy and Wolff-Parkinson-White. The variant has not been reported in Latin America; hence, this is the first report of the Arg302Gln mutation in the PRKAG2 gene in mestizo Ecuadorian subjects with mainly European ancestry components.

## Introduction

Genomic screening is an informative and helpful tool for the clinical management of inherited conditions such as cardiac diseases. Thus, the results could be essential for identifying the disease causative, starting the patient early and targeting treatment, and increasing the chances of survival since there is evidence of an increased risk of sudden death in some cardiac conditions (1).

Cardiac-inherited diseases are a group of disorders affecting the heart, its system, function, and vasculature (2). In 2015, the % of deaths caused by heart disease was 20.3% for the Hispanic ethnic group in the United States (3). In Ecuador, in 2016, the first cause of death was acute myocardial infarction which constituted 9.05% of deaths (4).

Among the cardiac inherited abnormalities, one of the most common is Wolff-Parkinson-White syndrome (WPW), an autosomal dominant disease; its prevalence fluctuates between 0.68 and 1.7/1000 (5). WPW involves an abnormal atrioventricular electrical conductive with an electrocardiogram (ECG) pattern of short PR interval and prolonged QRS (6, 7). Its associated symptoms include tachycardia, such as palpitations, syncope, dizziness, and sudden death (7).

Furthermore, hypertrophic cardiomyopathy (HCM) is one of the most common autosomal dominant inherited cardiac diseases, with an estimated prevalence of 1 per 200 to 500 people in the general population (8). Recent studies suggest that approximately 20 million people worldwide are affected by HCM (just 10% of cases are identified) (8–10). HCM diagnosis is based on the presence of a non-dilated left ventricular hypertrophy, identified by an echocardiogram or magnetic resonance (8). At a cellular level, cardiac myocytes are hypertrophied, disorganized, and separated by fibrosis areas (11).

The HCM is associated with an increased incidence of WPW syndrome; reports have suggested that it could be caused by a mutation in the protein-coding gene PRKAG2, which encodes a subunit of the AMP-activated protein kinase (AMPK). AMPK is essential in regulating cellular ATP energy metabolism since it activates energy production and inhibits energy consumption (5, 12).

Here we report a complete genomic screening for cardiac inherited conditions of two relatives to improve early diagnosis of familial cases.

## Case description

### Clinical phenotype

**Figure 1:**
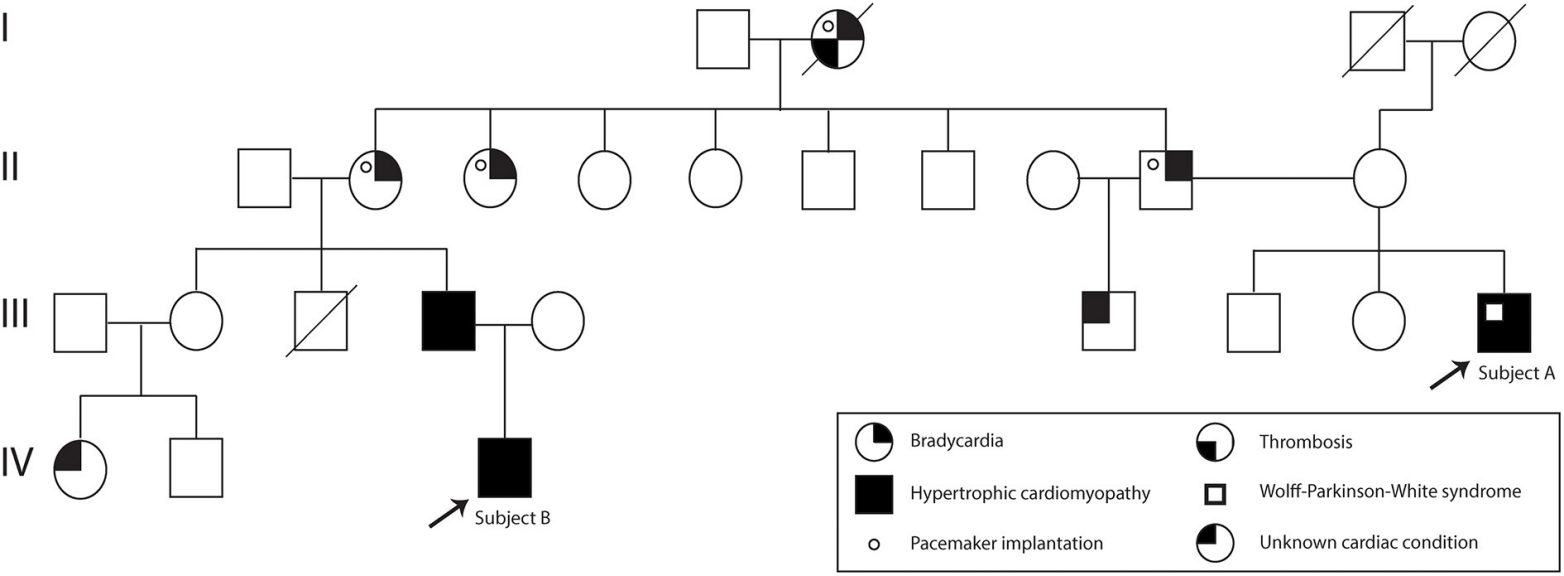
Subject A and B pedigree. Subject A and Subject B are indicated with an arrow. The family history of cardiac problems is depicted in the pedigree.

A 37-year-old Ecuadorian male (Subject A) with familiar history of bradycardia; his father, two paternal aunts, and paternal grandmother underwent cardiac pacemaker implantation because of the bradycardia. Additionally, a cousin (father of subject B) was diagnosed with hypertrophic cardiomyopathy, and a half-brother from his father's side suffers an unknown cardiac condition (Figure 1). The patient went to the physician at the first time when he was 23 years old because of symptoms such as tachycardia, dizziness, shortness of breath, and a feeling of fainting at any time of the day. He was referred to the cardiologist, and electro and echocardiogram were performed. The diagnoses of WPW and HCM were based on the electrocardiogram, which showed a sinus rhythm with a short P-R interval accompanied by a prolonged QRS complex with a delta wave. A complete right bundle branch block with a significant increase in R wave voltages in the anterior, septal, low lateral, and high lateral (DI and aVL) regions was observed. A deep T wave inversion was identified in this same plane, and a low level in the segment ST-T V3-V6 (Supplementary Figure S1). The echocardiogram confirmed a structurally normal heart. Carvedilol was initially prescribed at a dose of 6.25 mg per day. However, four months prior to the publication of this case report, the dose increased to 12.5 mg daily.

Fourteen years later after the initial diagnosis, a genomic test was performed.

**Figure 2:**
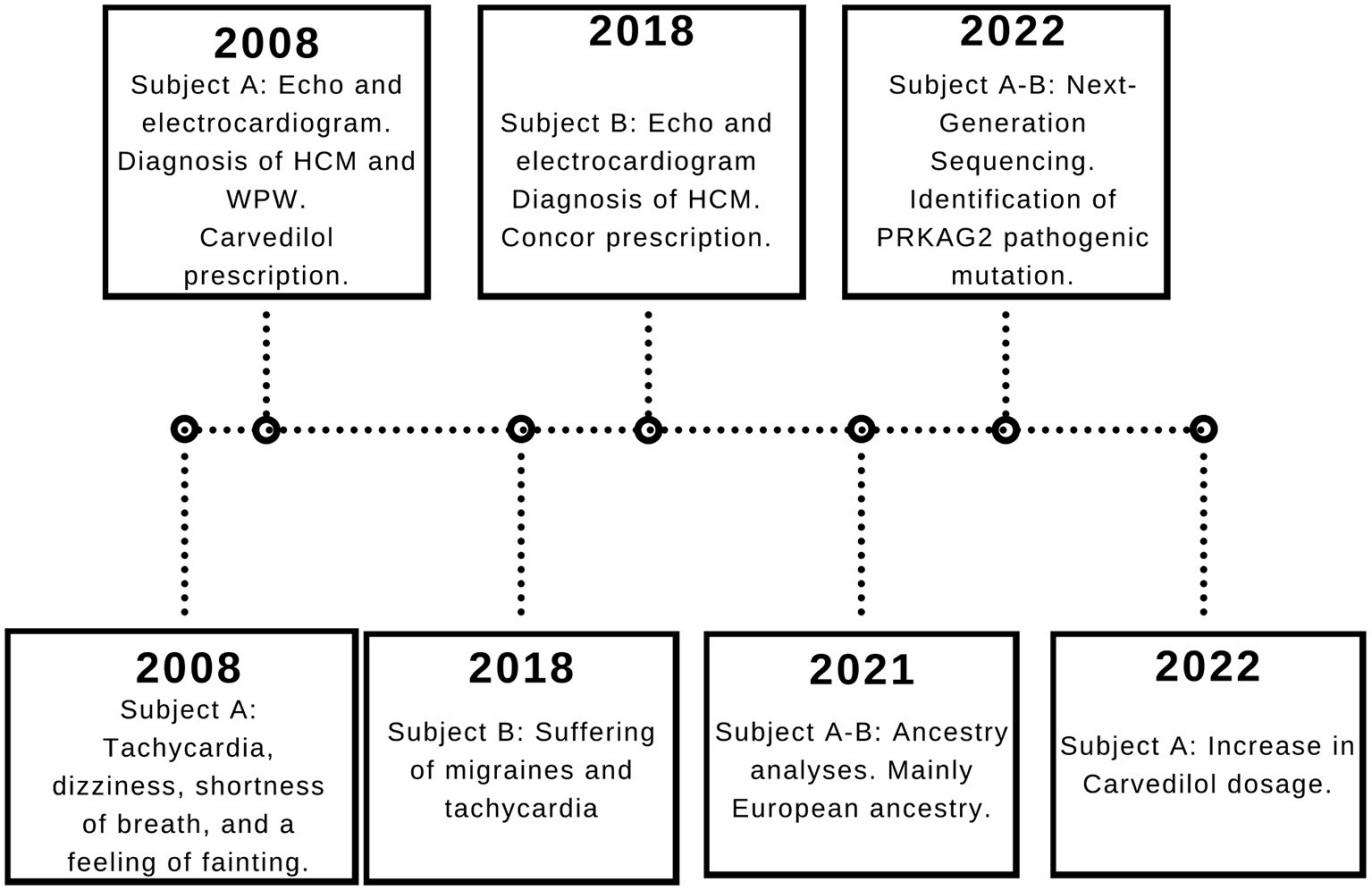
Subject A and B timeline. The relevant data is displayed in the timeline.

Moreover, 3 years ago, his cousin's son, an 18-year-old Ecuadorian male (Subject B) with the same familiar history of cardiac conditions (Figure 1), was referred to the cardiologist after suffering from migraines and tachycardia at any time of the day, especially after a sudden movement. Subject B underwent an electrocardiogram, and the results showed a systolic overload compatible with hypertrophic myocardiopathy, no association with WPW was found. Bisoprolol was prescribed at a dose of 1.25 mg daily, and the subject reported a significant reduction in the frequency of migraines. Similar to Subject A, a genomic screening was done. The timeline regarding subject A and B is presented in Figure 2.

### Sampling and DNA extraction

Patients A and B self-identified as “mestizo” and signed the informed consent to participate in the study.

A peripheral blood sample was collected in an EDTA tube. The DNA was extracted with PureLink Genomic DNA Mini Kit (Life Technologies) according to the manufacturer's protocol (13).

The DNA quantity and purity were measured by spectrophotometry and fluorometric methods; the quality was analyzed with an agarose gel. The sample was then diluted to 5ng/μL according to the sequencing protocol.

### Next-generation sequencing (NGS)

The genomic procedure was performed at Centro de Investigación Genética y Genómica, using the commercially available kit TruSight Cardio kit (Illumina), covering 174 genes (575 kb of cumulative target region size) with known association with 17 Inherited Cardiac Conditions (47 genes for Hypertrophic Cardiomyopathy). The samples were processed according to the manufacturer's protocol (14) and run for 315 cycles in MiSeq System (Illumina).

### Ancestral proportion analysis

For ancestral proportion analyses, PCR amplification was done with 46 ancestral informative INDEL markers in one multiplex reaction, according to Pereira et al. (15). Fragment separation and detection were executed on the 3,500 Genetic Analyzer (Thermo Fisher Scientific). The results were collected on Data Collection v 3.3 and analyzed in Gene Mapper v. 5 (Thermo Fisher Scientific).

### Genomic and statistical data analysis

Genomic data analysis was done following the pipeline: 1) the alignment to the reference genome (hg38) and variant caller with DRAGEN Enrichment v. 3.9.5; 2) the annotation with Annotation Engine v.3.15; 3) the consequence prediction by PolyPhen and Sift; 4) the variants were filtered in the software Variant Interpreter v.2.16.1.300

The ancestral inference analysis was done with STRUCTURE v.2.3.4 using the option for an admixture model.

## Diagnostic assessment

### Genomic results

After applying variant filters (pass quality and association including pathogenic, likely pathogenic, and of uncertain significance), nine variants were displayed to be involved in the diagnosis of subject A; however, two intronic and two PolyPhen-predicted benign variants were discarded. The disease-relevant variants are presented in Table 1.

**Table 1:** Genomic variants found in Subject A.

**Gene**	**Variant**	**Consequence**	**Association**	**Zygosity**
PRKAG2	SNV	Missense	Pathogenic	Heterozygous
	c.905G>A			
	p.(Arg302Gln)			
GCKR	SNV	Missense	Likely Pathogenic	Heterozygous
	c.307G>A			
	p.(Val103Met)			
APOB	SNV	Missense	Variant of uncertain significance	Heterozygous
	c.9871C>T			
	p.(Arg3291Cys)			
TTN	SNV	Missense	Variant of uncertain significance	Heterozygous
	c.44986C>T			
	p.(Arg14996Cys)			
PKP2	SNV	Missense	Variant of uncertain significance	Heterozygous
	c.1726A>T			
	p.(Met576Leu)			

After applying variant filters (pass quality and association including pathogenic, likely pathogenic, and of uncertain significance) in subject B, six variants were found; however, one intronic and three predicted as benign by PolyPhen were discarded. The two diagnosis-associated variants are presented in Table 2.

**Table 2:** Genomic variants found in Subject B.

**Gene**	**Variant**	**Consequence**	**Association**	**Zygosity**
PRKAG2	SNV	Missense	Pathogenic	Heterozygous
	c.905G>A			
	p.(Arg302Gln)			
TTN	SNV	Missense	Variant of uncertain significance	Heterozygous
	c.8056A>G			
	p.(Ile2686Val)			

Both individuals presented the same mutation c.905G>A p.(Arg302Gln) in the gene PRKAG2, which has been associated with PRKAG2 cardiac syndrome.

### Ancestral characterization

Subject A has a major European (51%) composition, followed by Native American (43.9%) and African (5.1%).

Moreover, subject B also has a major European (51.1%), followed by Native American (41.6%) and African (7.3%) composition.

Data corroborates that both individuals are a population mixture, in agreement with their initial self-identification.

## Discussion

Nowadays, genetic testing is increasingly recommended, as it provides helpful information for clinical management, including early diagnosis, family screening, and targeted therapy for cardiovascular diseases. Although, NGS has limitations, such as the need for bioinformatic knowledge for sample processing, interpretation of variants of unknown significance, or management of incidental findings (16). The American Heart Association has encouraged research in cardiovascular genetics to further understand the molecular aspects of these diseases (17). In the present case report, the TruSight Cardio (Illumina) kit was used; the panel of this kit consists of 174 genes that have been associated with 17 different cardiovascular diseases. By analyzing this amount of information, the chances of identifying pathogenic or likely pathogenic variants associated with the clinical diagnosis increase.

Heart diseases are the most common cause of death with no correlation with demographic or ethnic factors (5, 11). Among cardiomyopathies, HCM is the most common condition (18). Sarcomeric protein mutations have been primarily associated with familial or sporadic cases of HCM; however, non-sarcomere proteins involved in biological processes such as cellular metabolism have also been correlated to HCM (19). Moreover, regarding WPW, about 2 to 5% of patients diagnosed with HCM exhibit WPW (11, 20, 21).

The serine/threonine protein kinase AMPK regulates cellular energetic homeostasis. The enzyme is stimulated by AMP concentration and the activity of the AMPK-kinase; its principal function is to counterbalance ATP depletion. Moreover, AMPK comprises three subunits (α, β, γ). The Gamma 2 regulatory subunit of AMP-Activated Protein Kinase (PRKAG2) activity enhances the Alpha subunit activation by binding to AMP (21, 22). Mutations in the gene that encodes PRKAG2 have been associated with glycogen storage cardiomyopathies by disrupting AMPK's ability to bind to AMP, dysregulating the metabolic and glucidic uptake, and causing the deposition of amylopectin and glycogen (5). The symptoms of the PRKAG2 cardiac syndrome vary, ranging from asymptomatic to a phenotype that includes cardiac hypertrophy, primarily involving the left ventricle, supraventricular tachycardia (WPW), increased risk of heart failure, and sudden cardiac death (5, 22). The results of NGS identified a pathogenic missense mutation G>A at position 905, causing a change from arginine to glutamine at codon 302, exon 7 of the PRKAG2 gene, in both subjects; however, only subject A presents WPW.

Similarly, different mutations in the gene TTN were found. In subject A, a missense mutation Arg14996Cys, and in subject B, a missense mutation Ile2686Val; however, both variants are categorized as variants of uncertain significance (VUS), and mutations in the TTN gene have been primarily associated with dilated cardiomyopathy, not hypertrophic (23, 24). Moreover, three more variants were found in subject A, two VUS in the genes APOB and PKP2, and one likely pathogenic variant in the gene GCKR; this variant has been associated with a higher risk of type 2 diabetes (25).

Ancestry analyses were performed to determine the genetic background of both subjects. Subjects A and B had mainly European ancestry components. Databases such as Allele Frequency Aggregator (26), The Exome Aggregation Consortium (ExAC) (27), and The genome Aggregation Database-Exomes (gnomAD-Exomes) (28) were used for information regarding the frequency, in Latin America, of the identified variant. The mutation has not been reported in Latin American populations; hence, this is the first report of an Arg302Gln mutation in the PRKAG2 gene in Latin America.

## Conclusion

Genetic testing is a useful tool as it can provide important information regarding a disease, including its cause and consequences, not only for single individuals but also to identify at-risk relatives. Furthermore, NGS results could guide the physician into targeted therapy. In the present case report, a missense pathogenic Arg302Gln mutation in the PRKAG2 gene has been identified in two related Ecuadorian Subjects diagnosed with hypertrophic myocardiopathy and Wolff-Parkinson-White. The variant has not been reported in Latin America; hence, this is the first report of the Arg302Gln mutation in the PRKAG2 gene in mestizo Ecuadorian subjects with mainly European ancestry components.

Unfortunately, in the presented family case, the access to other family members' samples were limited due to their refusal and unavailability to participate in the study.

## Data availability statement

The results are presented in the paper. Genomic Data are available in NCBI Sequence Read Archive (SRA) with the BioProject accession number PRJNA891265 (http://www.ncbi.nlm.nih.gov/bioproject/891265). For more information, please contact the corresponding author AZ (anazambrano17@hotmail.com).

## Ethics statement

The studies involving human participants were reviewed and approved by Comité de Ética de Investigación en Seres Humanos—Universidad UTE. The patients/participants provided their written informed consent to participate in this study.

## Author contributions

Conceptualization and formal analysis: SC-U and AZ. Resources: TS. Methodology: AZ, SC-U, PG-R, VR-P, EP-C, and RT-T. Writing—review and editing: AZ, SC-U, PG-R, VR-P, EP-C, ND, AI-R, and RT-T. Supervision, project administration, and funding acquisition: AZ. All authors contributed to the article and approved the submitted version.

## Funding

The project was funded by Universidad UTE.

## Conflict of interest

The authors declare that the research was conducted in the absence of any commercial or financial relationships that could be construed as a potential conflict of interest.

## Publisher's note

All claims expressed in this article are solely those of the authors and do not necessarily represent those of their affiliated organizations, or those of the publisher, the editors and the reviewers. Any product that may be evaluated in this article, or claim that may be made by its manufacturer, is not guaranteed or endorsed by the publisher.

## Supplementary material

Supplementary Figure S1 Subject A electrocardiogram.

## References

[1] Waddell-Smith KE Donoghue T Oates S Graham A Crawford J Stiles MK. Inpatient detection of cardiac-inherited disease: the impact of improving family history taking. Open Hear. (2016) 3:e000329. Available online at: http://openheart.bmj.com/26925241 10.1136/openhrt-2015-000329 PMC4762189

[2] NHS England. NHS Standard Contract for Cardiology: Inherited Cardiac Conditions. NHS Comm Board (2013). p. 1–22. Available online at: http://www.england.nhs.uk/wp-content/uploads/2013/06/d01-com-dis-equ-prosth.pdf

[3] Centers for Disease Control Prevention. Underlying Cause of Death, 1999-2020 Request. CDC WONDER Online Database (2020). Available online at: https://wonder.cdc.gov/ucd-icd10.html

[4] Ministerio de Salud Pública del Ecuador. Perfil de Mortalidad por Sexo 2016. (2017). Available online at: https://public.tableau.com/profile/darwin5248#!/vizhome/defunciones2016/Historia1?publish=yes

[5] Porto AG Brun F Severini GM Losurdo P Fabris E Taylor MRG. Clinical spectrum of PRKAG2 syndrome. Circ Arrhythmia Electrophysiol. (2016) 9:e003121. 10.1161/CIRCEP.115.003121 PMC4704128 26729852

[6] MacRae CA Ghaisas N Kass S Donnelly S Basson CT Watkins HC. Familial hypertrophic cardiomyopathy with Wolff-Parkinson-White syndrome maps to a locus on chromosome 7q3. J Clin Invest. (1995) 96:1216–20. 7657794 10.1172/JCI118154 PMC185741

[7] Gupta A Al-Ahmad A. Wolff parkinson white syndrome. In: Cardiac Electrophysiology: Clinical Case Review. StatPearls Publishing (2021). p. 267–9. Available online at: https://www.ncbi.nlm.nih.gov/books/NBK554437/

[8] Antunes M de O Scudeler TL. Hypertrophic cardiomyopathy. IJC Hear Vasc. (2020) 27:100503. 10.1016/j.ijcha.2020.100503 32309534 PMC7154317

[9] Maron BJ. Clinical course and management of hypertrophic cardiomyopathy. N Engl J Med. (2018) 379:655–68. 10.1056/NEJMra1710575 30110588

[10] Maron BJ Rowin EJ Maron MS. Global burden of hypertrophic cardiomyopathy. JACC Hear Fail. (2018) 6:376–8. Available online at:10.1016/j.jchf.2018.03.004 29724362

[11] Marian AJ Braunwald E. Hypertrophic cardiomyopathy: Genetics, pathogenesis, clinical manifestations, diagnosis, and therapy. Circ Res. (2017) 121:749–70. 10.1161/CIRCRESAHA.117.311059 28912181 PMC5654557

[12] Blair E Redwood C Ashrafian H Oliveira M Broxholme J Kerr B. Mutations in the gamma(2) subunit of AMP-activated protein kinase cause familial hypertrophic cardiomyopathy: evidence for the central role of energy compromise in disease pathogenesis. Hum Mol Genet. (2001) 10:1215–20. 10.1093/hmg/10.11.1215 11371514

[13] Life Technologies. PureLink^®^ Genomic DNA Kits for Purification of Genomic DNA. Invitrogen by life Technologies (2013). p. 1–48. Available online at: https://tools.thermofisher.com/content/sfs/manuals/purelink_genomic_man.pdf

[14] Illumina. Customize a short end-to-end workkow guide with the Custom Protocol Selector TruSight ^®^ Cardio Sequencing Kit Reference Guide. 2016;

[15] Pereira R Phillips C Pinto N Santos C dos Santos SE Amorim A. Straightforward inference of ancestry and admixture proportions through ancestry-informative insertion deletion multiplexing. PLoS ONE. (2012) 7:e29684. 10.1371/journal.pone.0029684 22272242 PMC3260179

[16] Di Resta C Galbiati S Carrera P Ferrari M. Next-generation sequencing approach for the diagnosis of human diseases: Open challenges and new opportunities. Electron J Int Fed Clin Chem Lab Med. (2018) 29:4–14. 29765282 PMC5949614

[17] Musunuru K Hershberger RE Day SM Klinedinst NJ Landstrom AP Parikh VN. Genetic testing for inherited cardiovascular diseases: A scientific statement from the american heart association. Circ Genomic Precis Med. (2020) 13:E000067. 10.1161/HCG.0000000000000067 32698598

[18] Heron M. National Vital Statistics Reports Volume 68, Number 6, June 24, 2019, Deaths: Leading Causes for 2017. (2019). Available online at: https://www.cdc.gov/nchs/products/index.htm 32501203

[19] Elliot PM Zamorano JL Anastasakis A Borger MA Borggrefe M Cecchi F. 2014 ESC guidelines on diagnosis and management of hypertrophic cardiomyopathy: The task force for the diagnosis and management of hypertrophic cardiomyopathy of the European Society of Cardiology (ESC). Eur Heart J. (2014) 35:2733–79. 10.1093/eurheartj/ehu284 25173338

[20] Kelly BS Mattu A Brady WJ. Hypertrophic cardiomyopathy: electrocardiographic manifestations and other important considerations for the emergency physician. Am J Emerg Med. (2007) 25:72–9. 10.1016/j.ajem.2006.04.017 17157688

[21] Aggarwal V Dobrolet N Fishberger S Zablah J Jayakar P Ammous Z. PRKAG2 mutation: An easily missed cardiac specific non-lysosomal glycogenosis. Ann Pediatr Cardiol. (2015) 8:153–6. 10.4103/0974-2069.154149 26085771 PMC4453188

[22] Banankhah P Fishbein GA Dota A Ardehali R. Cardiac manifestations of PRKAG2 mutation. BMC Med Genet. (2018) 19:17–20. 10.1186/s12881-017-0512-6 29298659 PMC5751825

[23] Begay RL Graw S Sinagra G Merlo M Slavov D Gowan K. Role of titin missense variants in dilated cardiomyopathy. J Am Heart Assoc. (2015) 4:1–9. 10.1161/JAHA.115.002645 26567375 PMC4845231

[24] Norton N Duanxiang L Rampersaud E Morales A Martin ER Zuchner S. Exome sequencing and genome-wide linkage analysis in 17 families illustrates the complex contribution of TTN truncating variants to dilated cardiomyopathy. Circ Cardiovasc Genet. (2013) 6:1–7. 10.1161/CIRCGENETICS.111.000062 23418287 PMC3815606

[25] Rees MG Ng D Ruppert S Turner C Beer NL Swift AJ. Correlation of rare coding variants in the gene encoding human glucokinase regulatory protein with phenotypic, cellular, and kinetic outcomes. J Clin Invest. (2012) 122:205–17. 10.1172/JCI46425 22182842 PMC3248284

[26] Phan L Jin Y Zhang H Qiang W Shektman E Shao D. ALFA: Allele Frequency Aggregator. National Center for Biotechnology Information; U.S. National Library of Medicine (2020). Available online at: http://www.ncbi.nlm.nih.gov/snp/docs/gsr/alfa

[27] Karczewski KJ Weisburd B Thomas B Solomonson M Ruderfer DM Kavanagh D. The ExAC browser: Displaying reference data information from over 60 000 exomes. Nucleic Acids Res. (2017) 45:D840–5. 10.1093/nar/gkw971 27899611 PMC5210650

[28] Karczewski KJ Francioli LC Tiao G Cummings BB Alföldi J Wang Q. The mutational constraint spectrum quantified from variation in 141,456 humans. Nature. (2020) 581:434–43. 10.1038/s41586-020-2308-7.. 32461654 PMC7334197

