# Supplementary figures and images for: Case report: Genomic screening for inherited cardiac conditions in Ecuadorian mestizo relatives: Improving familial diagnose
Source: Front Cardiovasc Med. 2022 Nov 8;9:1037370. doi: 10.3389/fcvm.2022.1037370 (PMC9678921; doi:10.3389/fcvm.2022.1037370)

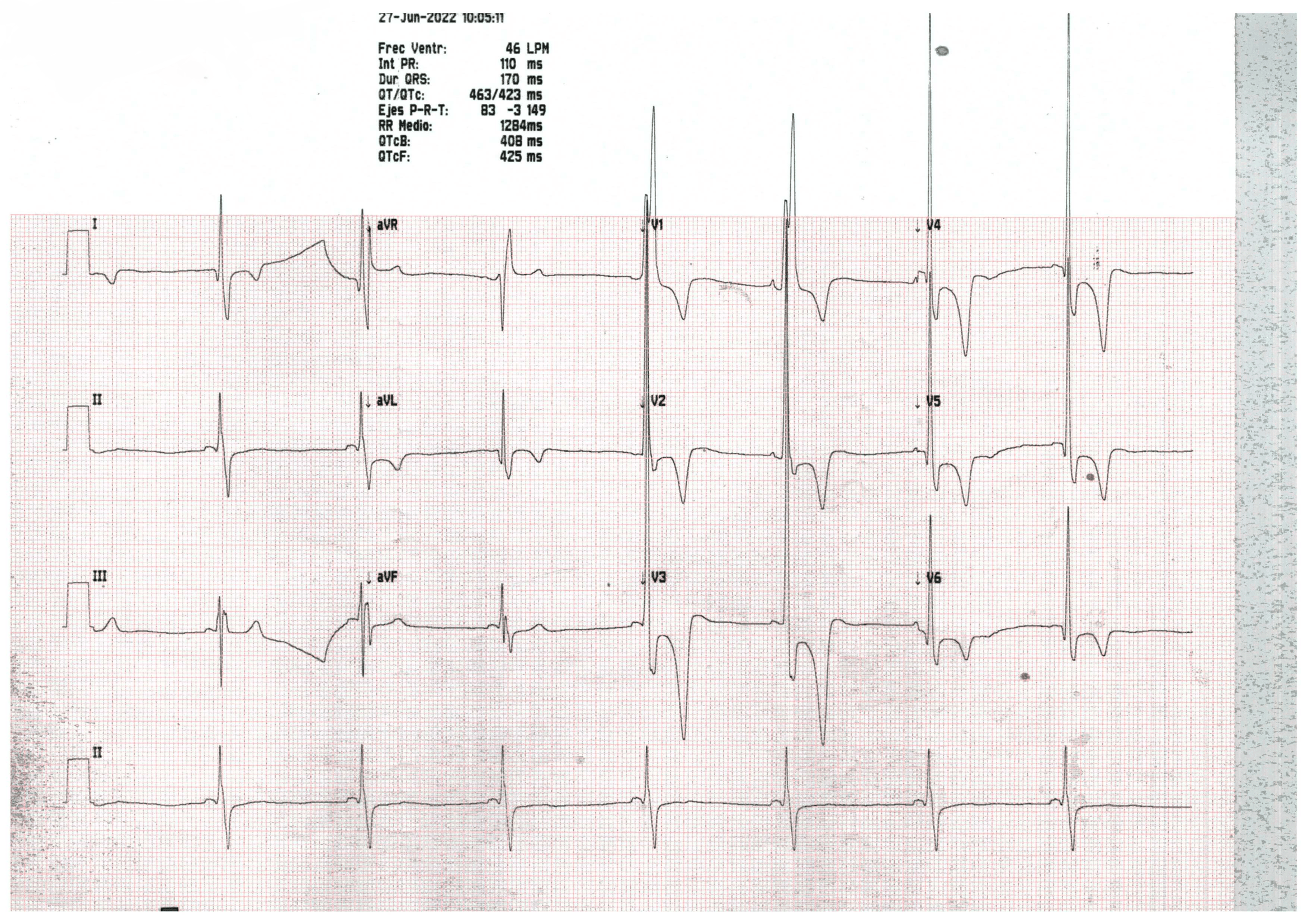

Supplement: Supplementary Figure S1 — Subject A electrocardiogram. [file Image_1.PNG]
